# Impact of R&D innovation and political background on corporate growth: A study based on private listed companies in China

**DOI:** 10.1371/journal.pone.0297329

**Published:** 2024-05-09

**Authors:** Xiangde Kong, Hongan Chen, Peng Wu, Ran Ma, Fei Pan

**Affiliations:** 1 School of Business, East China University of Science and Technology, Shanghai, China; 2 School of Business, Yangzhou University, Yangzhou, Jiangsu, China; 3 Civil and Commercial Law School, Shandong University of Political Science and Law, Jinan, Shandong, China; 4 School of Business, University of Shanghai for Science and Technology, Shanghai, China; Lingnan University, The University of HongKong, HONG KONG

## Abstract

Based on a review of related concepts and theories this study investigates the different impacts of research and development (R&D) innovation and political background on corporate growth in a particular context. Unlike other studies, we integrate these two factors. We empirically analyze 6079 sets of data from 1292 A-share private manufacturing enterprises in Shanghai and Shenzhen from 2012 to 2019. The results show that these factors directly impact corporate growth and have heterogeneous effects at different enterprise growth levels. We find the effect of R&D innovation on corporate growth is more pronounced for young firms. These findings highlight the need for firms to adjust their investments in R&D innovation and political backgrounds at different stages of development to adapt to different markets and political environments.

## 1. Introduction

Following China’s reform and opening-up, the rapid growth of its economy led to economic development and the rapid growth of key local corporations. Investments in resources were made across the board to promote corporate research and development (R&D) innovation and many preferential policies were introduced to support and encourage corporate innovation. However, the results of this innovation did not fundamentally change. Although China’s economic aggregate and household income significantly increased, China has also seen the emergence of companies such as Huawei, which has leapfrogged the Swedish giant Ericsson and become a case of global market leaders, government policies have played an important role in this process [1, 2], but many local Chinese enterprises still lacked core competitiveness. We question why has China not created many core competitive local corporations, despite its rapid economic development. We surmise that other factors besides China’s unique political and economic environment and core competitiveness (based on R&D innovation ability) could effectively promote corporate growth and performance improvement too. These questions have attracted the interest of several researchers.

Through R&D innovation, corporations can produce new products, invent new technologies, and update business models. They can take the lead in accumulating market experience, occupy a favorable market position, and form entry barriers for potential entrants striving for a more significant market share and super profit [3].

Most extant studies analyze the impact of R&D innovation or political background on corporate growth from a single perspective; however, few studies put these two factors into the same framework to analyze their influence mechanisms and integration effects on corporate growth. Unlike other studies, we integrate these two factors. China’s economy was in a transitional period, which was of great significance for local corporations seeking corporate growth in this particular period. According to the theory of core competitiveness, corporate R&D innovation ability is the root of core competitiveness and key to sustainable development. Numerous studies have shown that R&D innovation is conducive to higher performance and faster corporate growth. However, when it comes to competitive advantages that promote corporate growth, many local entrepreneurs do not want to improve their core competencies by investing in risky, slow, and long-term R&D projects, but prioritize the ability to integrate resources. Political background is undoubtedly an effective means for corporations to quickly acquire this capability. During the Chinese economic transition, many entrepreneurs actively ran for National People’s Congress (NPC) deputies or Chinese People’s Political Consultative Conference (CPPCC) members and hired government officials to work in their corporations. Thus, there were significant differences between the theoretical research and corporate practice regarding the core competencies required for the growth of Chinese corporations. Based on this, this study attempts to deeply analyze the role of R&D innovation and political background in the development of local Chinese corporations to illuminate the development of Chinese corporations.

Combining the specific characteristics of high and low growth rates, this study first investigates the heterogeneous effects of political backgrounds and R&D innovation on corporate growth at different growth rates. Second, it examines the moderating effects of corporate age and the choice between R&D innovation and the political background of local Chinese corporations in the corporate development process and proposes some suggestions. In this study, 2012–2019 Shanghai and Shenzhen A-share private manufacturing listed companies were selected as the sample data to test the hypothesis. Referring to the relevant studies on endogeneity [4, 5], we take the natural logarithm of the relevant variables and one stage lag to solve the nonlinear relationship that may occur in the data analysis process.

## 2. Literature review and research hypothesis

### 2.1 Average impact of R&D innovation on corporate growth

[6] noted that when the external economic environment fluctuates widely, inferior and obsolete products/services are eliminated from the market, whereas high-quality innovative products/services continue to survive. Consumers become more price-sensitive and cautious when making purchasing decisions during economic fluctuations. Innovative products or services are more technologically advanced, of higher quality, and have higher cost performance to provide a higher consumer surplus; therefore, consumers are more likely to choose innovative products and services [7]. Moreover, an improvement in R&D innovation capability provides a comparative advantage for corporations to translate new knowledge into a higher market share [8]. On the one hand, the "learning by doing" effect enables corporates to accumulate technical experience continuously [9], which is conducive to incubating new knowledge or skills internally through R&D innovation activities. On the other hand, improving R&D ability can improve corporations’ efficiency in absorbing the innovation results of other organizations and saving on R&D costs, thus increasing their ability to resist risks.

Additionally, increasing investments in innovation increases the possibility of improving corporate growth, which can promote the development of diversified corporations. This improves their relative competitiveness and promotes growth. Research has also shown that increasing investment in innovation can help corporations improve production efficiency [10], increase their total output, and seek scale advantages [11].

However, translating innovative activities into market share requires significant investments in time and resources, and the innovation process is characterized by long-duration cycles and high uncertainty [12]. Thus, R&D innovation inhibits corporate growth. First, companies engaged in R&D innovation do not necessarily achieve higher profits or growth rates. For example, some traditional private corporations have less access to technological innovation. Their R&D innovations do not then constitute entry barriers for competitors, which reduces their profits and competitive advantages. Second, some corporations are affected by many factors, such as imperfect operation mechanisms, leaders’ weak innovation consciousness, and action forces. A large proportion of many corporations’ R&D investment are sunk costs as they do not produce substantial results. This hinders their growth. Third, due to the limitations of the law of technological evolution, R&D projects with high expectations are affected by long cycles and limited capital input, and the output of R&D innovation achievements is slow. Serious transformation difficulties enhance the low efficiency of corporate R&D innovation. Corporations may suffer economic losses owing to the failure to recover previous investments smoothly. Thus, a simple increase in R&D intensity does not necessarily lead to corporate growth. In addition, from the perspective of new and old products, the statistical relationship between R&D innovation and corporate growth can be negative. This is when the firm ceases to provide the old product or service but the new one cannot successfully replicate the previous market performance. This negative relationship is stronger when the technological level of the industry in which the firm operates changes rapidly and its R&D level cannot keep up with its competitors. If a corporation continues to provide old products or services while launching new products or services, both will occupy each other’s market share. This makes the corporation its own competitor, which may lead to a decline in overall performance.

The above discussion indicates that R&D innovation may not only promote the growth of corporations, but also restrain their growth. Therefore, it is necessary to further analyze the mechanism of R&D innovation based on the specific situation of Chinese corporations. To maintain steady growth, Chinese corporations must cultivate and develop their innovation abilities through R&D to improve their market share and competitiveness.

Based on the above analysis, this paper proposes the following fundamental research hypotheses:

H1: An increase in R&D innovation input is conducive to corporate growth.

### 2.2 Differentiated impact of R&D innovation on corporate growth

The inherent attributes of R&D innovation make it subject to financing constraints. Owing to the nature of intangible assets, R&D investments cannot be mortgaged. Simultaneously, information asymmetry causes investors to face adverse selections with more significant risks; thus, they are cautious when choosing whether to invest. In addition, competitors’ imitation reduces expected returns on R&D, which further increases uncertainty [13, 14]. Many studies have shown that financing constraints crucially impact R&D innovation. When corporations have insufficient working capital and limited external financing, their R&D costs are higher and they choose to cease innovation or reduce their R&D investment [15, 16]. [17] noted that financial barriers have a more significant marginal impact on firms’ innovation choices than other non-financial barriers (e.g., demand uncertainty generated by market barriers) and knowledge barriers (e.g., a lack of adequate technical information). Therefore, corporations face significant financing constraints when conducting R&D innovation projects. Simultaneously, the rapid economic growth in emerging markets has spawned many market opportunities, leading corporations to pursue faster growth and higher profits. With their rapid growth, corporations have gained legitimacy, popularity, and market power [18]. In addition, the rapid growth of corporations in emerging markets is essential for attracting critical external resources. Owing to the different internal characteristics of corporations, those with high and low growth rates face different degrees of financing constraints. Corporations with low growth rates show a strong tendency to sell productive assets and give up some investment projects [19]. In comparison, corporations with high growth rates have more development potential, have a stronger ability to grasp investment opportunities, face lower financing constraints [20], and can reduce the negative impact of financing constraints on R&D innovation.

Second, from the perspective of knowledge capital, high-growth firms tend to possess more relevant and valuable knowledge, resources, and social capital than low-growth/negative-growth corporations when all else is equal [21]. Therefore, compared with low-growth firms, high-growth firms are more likely to achieve innovation success based on internal higher-value knowledge, resources, and networks.

Finally, from the employee perspective, corporate growth provides more promotion opportunities, higher salaries, and prestige. At the same time, as companies "break out of the box" and expand into new business areas, employees’ work becomes more challenging, which greatly stimulates the enthusiasm of employees to innovate. By contrast, corporations with stagnant growth or even retrogression form a rigid internal environment lacking innovation awareness, significantly reducing innovation efficiency and intention [22].

Corporations at different growth levels face significant differences in internal and external resources, which are suitable for different development paths and strategies. This study argues that the role of R&D innovation in different growth stages of corporations is constantly evolving, and its marginal effect should be dynamic. Accordingly, the following research hypothesis was proposed:

H2: R&D innovation has a heterogeneous effect on corporate growth. Compared with low-growth corporations (corresponding to low score locus), R&D innovation has a stronger promotional effect on the growth of high-growth corporations (corresponding to high score locus).

### 2.3 Influence of political background on corporate growth

In the context of China’s system, local government officials have significant power because of their control of preferential policies or economic resources, such as administrative approval and loan guarantees [23]. Therefore, an increasing number of corporations seek to establish political background [24]. [25] found that private Chinese corporations tend to gain government support by establishing close relationships with the government to accelerate their growth. The promotional effect of political background on corporate growth includes the following aspects.

First, political background can be used as alternative financial mechanisms to provide financing facilities for corporations and meet their capital needs for long-term sustainable development [26]. Second, when the regional tax burden is relatively heavy, corporations with political backgrounds have more opportunities to reduce taxes and increase investor profits, attracting more investment and promoting corporate growth [27]. Third, politically connected firms are more likely to receive government funding than non-politically connected firms. Politically connected firms are also more likely to receive funding from the IMF or the World Bank than non-politically connected firms [26]. Fourth, corporations with political backgrounds are more likely to consider government procurement. Government procurement can provide long-term and stable financial support for corporations and promote their growth. Fifth, in transition economies, private Chinese enterprises tend to build political backgrounds with the government. They can take advantage of the inherent advantages of government officials’ political identities to reduce the difficulty in protecting their property rights through formal institutions [28]. Government policies are undoubtedly the most important external factors affecting corporate development. Politically connected corporations can grasp policy orientation and change earlier; hence, they have more time to allocate resources and capabilities in advance, maximize the use of their advantages, and avoid policy risks that are not conducive to corporations. In some industries that involve a monopoly of state-owned enterprises, access qualifications can be obtained quickly and efficiently by virtue of the political backgrounds of government officials, thus promoting their growth. This verifies the alternative mechanism proposed by [29] to achieve the full development of the financial market and economy for Chinese investors under the condition of insufficient legal and market system protection. This increased the diversification of business operations and promoted corporate growth.

However, political associations may be achieved at the cost of corporations’ efficiency. First, the closer the relationship between the government and corporation, the more energy corporate executives must spend to maintain it. Second, the long-term reliance on non-market mechanisms to obtain development resources weakens corporations’ incentives for technological and management innovation, leading to low production and operational efficiency [30]. Finally, corporations may be forced to participate in political competition among government officials to cater to their political goals, thereby increasing costs and burdens. Compared with non-politically connected corporations, connected ones (under government performance pressure) need to take on more social responsibilities, so their business performance may be worse than that of non-politically connected companies [31].

The above analysis shows that the political background plays both supportive and predatory roles in corporations’ development processes. While it provides enterprises with government subsidies, tax incentives, industry access qualifications, and other support, it also interferes with the regular operation of corporations through rent-seeking, weakening their value and hindering their growth.

From a resource-dependence perspective, corporations’ growth rates are significantly affected by their ability to obtain external resources. Compared with those with low growth (negative growth), high-growth corporations are more dependent on external resources, suffer a more significant impact when the external market fluctuates wildly, and face financial crises. Thus, the uncertainty of high-growth corporations is also higher, and high corporate growth cannot be equated with high corporate value [32]. Thus, building a political background is one way for high-growth firms to stabilize their access to external resources and reduce operational uncertainty.

From the cost perspective of constructing political associations, high-speed growth corporations gain legitimacy, popularity, and market power. They can produce more positive social benefits, such as providing a large number of new employment opportunities, promoting regional economic growth, driving the rapid development of industry innovation and knowledge, and playing a leading and exemplary role in potential entrepreneurial corporations (achieving high growth, especially in terms of sales and market share growth), which is considered one of the most important goals of entrepreneurship orientation [33], the social benefits mentioned above can help local governments share part of the pressure of economic growth, and help government officials stand out in the promotion competition. In this case, local governments will more actively support the development of high-speed growth corporates, and in order to maintain the current development trend of high-speed growth corporates, they will not interfere too much in their management and operation, which will significantly reduce the cost of corporates to actively build political background, so that political background is more reflected as a “helping hand.” Compared with high-speed growth corporates, low/negative growth corporates need to pay higher construction costs, and regular operation will be too much interference by the government, the cumbersome “political burden,” and even lose the right to make independent decisions, hindering the sustainable growth of corporates. Given low-growth firms play a limited role in the performance evaluation of government officials and do not produce many positive social benefits (such as providing many new jobs), government officials will not take the initiative to provide such corporations with policy funds and other support, which requires corporations to invest more resources in building political backgrounds. Not only will a large number of rent-seeking costs significantly reduce the investment-to-income ratio, but they will also cause resource crowding out of productive activities such as research and development and innovation, thus seriously hindering the sound growth of corporations. Even if corporates with low/negative growth can successfully build political background and obtain some resources from the government, given their poor development status, the government will interfere to a large extent in the business decision-making of corporates in order to ensure the efficiency of resource use, hindering the regular operation of corporates with low/negative growth, making political background more reflected as “predatory hand.”

Therefore, corporations must consider the advantages and disadvantages of their political backgrounds during different development periods and develop political strategies that are suitable for corporate development. Based on the above analysis, this study proposes the following hypothesis:

H3: Political background has a heterogeneous effect on corporate growth, promoting the growth of high-growth firms (corresponding to high score locus) while inhibiting the growth of low-growth firms (corresponding to low score locus).

## 3. Research design

### 3.1 Research samples and data sources

State-owned enterprises represent national interests and cannot fully reflect the operation law of market economy [34]. To verify our hypothesis, A-share private manufacturing listed corporates on the Shanghai and Shenzhen Stock exchanges from 2012 to 2019 were selected as the initial samples. Samples of state-owned corporations at the time of listing and later becoming private (due to equity transfers) were excluded. Only private samples of corporations were retained because their listings remained unchanged. With the implementation of a series of policy activities such as "eight-point regulations and six-point bans" in China, the cost for corporations to establish political backgrounds is increasing. Additionally, considering that corporations have been significantly affected by COVID-19 since 2020, data after 2020 were not included in the sample scope of this study. The main reason Private corporations were selected as the research sample in this study because, compared with state-owned corporations, private corporations lack the natural advantages of political associations. They have a stronger motivation to obtain support and protection than they can from the formal system with the help of a political association, place the political association in a more prominent position, and invest more resources in constructing political associations. Thus, the influence of political backgrounds on corporate growth is more prominent among private corporations. Manufacturing corporations were selected for this study because the number of manufacturing samples was relatively large for all industry categories of listed corporations. Given that the manufacturing industry has been listed for the longest time, the data in its annual reports are more accurate and reliable. Thus, this study frames the research samples as listed corporations in the manufacturing industry.

To ensure the validity of the samples and robustness of the research results, the following screening was conducted on the basis of the initial samples: (1) remove the samples categorized as ST, *ST, and S; (2) exclude the samples issued by B shares and H shares at the same time; (3) exclude the samples with the political background of senior executives and the absence of R&D input data; (4) exclude the samples with outliers and missing values in financial indicators; (5) eliminate the samples of significant business changes and material asset reorganization during the sample period; (6) eliminate the samples listed less than one year. After screening, we collated 6,079 datasets from 1,292 corporations between 2012 and 2019.

The financial data used in this study were obtained from the CSMAR database and sorted. For the acquisition of data related to political associations, this study first obtains all senior executives’ names from the annual reports of corporations and then searches for the time when senior executives held government posts on the Internet. For some uncertain samples, we first log onto the website of the National People’s Congress or the Chinese People’s Political Consultative Conference (CPPCC) of the city where the enterprise is registered and compare the names of senior executives with the names of deputies to the National People’s Congress or CPPCC members. Second, this study used in-depth information on senior executives from the database for detailed verification. This method is more accurate than one that relies only on data from a database to define political associations. The specific raw data are shown in S1 File.

### 3.2 Definition of variables

#### 3.2.1 Dependent variable

*Corporate growth*. The existing research primarily uses comprehensive and single indices to measure corporate growth. The comprehensive index uses a hierarchical analysis, principal component analysis, factor analysis, and other methods to calculate the total score. However, a comprehensive index system is generally complicated, which can easily lead to data redundancy, and the setting of index weights is subjective, resulting in inaccurate calculations. In contrast, a single indicator selects a particular financial indicator to measure corporate growth. The most commonly used single indicators include Tobin’s Q, growth rate of the number of employees, growth rate of total assets, growth rate of net profit, and growth rate of primary business revenue. When Tobin’s Q is selected, the corporation must exist in a solid and efficient capital market in which investors are rational and stocks can circulate freely. Clearly, this is more suitable for developed capital markets. However, China’s capital markets need to be improved. Many domestic investors have speculative attitudes, resulting in significant stock market volatility and weak capital markets. Additionally, restricted stocks are common in the Chinese market. Therefore, it is inappropriate to use Tobin’s Q to measure local corporations growth. Regarding the growth rate of the number of employees and the growth rate of total assets, the factor intensities of different industries, or even among corporations in the same industry, are significantly different and constantly changing. Therefore, the regression results obtained using these two indicators do not reflect the growth of corporations but rather the change in corporate factor intensity. The net profit growth rate represents the change in corporate profitability and does not effectively reflect the overall picture of corporate growth. Based on the reality that China is in economic transition, this study draws on domestic scholars’ research and uses the operating income growth rate to measure corporate growth [35], named "*Growth*".

#### 3.2.2 Independent variable

*R&D innovation*. Existing studies use R&D investment intensity and total R&D investment to measure corporate R&D innovation behavior. Among these, total R&D investment is easily affected by firm size heterogeneity and does not truly reflect the matching relationship between R&D innovation and corporate growth. Therefore, based on [36], this study selected the intensity of R&D investment to measure corporations’ R&D innovation behavior and named it *R&D*. R&D investment intensity is expressed as the proportion of R&D expenditures to a corporation’s main business income. As a robustness test, the ratio of R&D personnel to the total number of employees is used to measure the intensity of R&D investment, named *R&D Per*.

*Political background*. Existing literature has different ways of measuring corporate executives’ political backgrounds. The dummy variable method has the highest universality among the measurement methods for political background. In recent years, scholars have assigned the level of political background to senior executives based on dummy variables to describe China’s unique institutional background more accurately. Scholars have also studied political backgrounds from the perspectives of chairpersons, general managers, local governments, and other objects. S2 File systematically combs the methods used to measure corporate executives’ political backgrounds and the types of variables used.

Based on current research results, this study sets corporations’ political backgrounds as dummy variables and names them *Political*. Suppose senior executives of corporations serve as government officials, including central or local party committees, procuratorates, courts, governments, people’s congresses, and the CPPCC. If the senior executive is currently or once served as a party representative, NPC deputy, or CPPCC member when one of the above conditions exists, the corporation has a political association, and the value is assigned a value of 1; otherwise, the value is assigned a value of 0. As a robustness test based on the evaluation method, this study considers the present or past political identity of corporate executives as the criterion for measuring political background, and assigns the political identity of corporate executives according to the administrative level. For the political associations of government officials (corporate executives used to work in government agencies), the evaluation criteria referred to their highest position levels: section level below 1, section level 2, division level 3, department level 4, and ministry level 5. For the political association of deputies and members (corporate executives are or are party deputies, NPC deputies, or CPPCC members), the value is assigned according to level: 1 at the township level, 2 at the county level, 3 at the city level, 4 at the provincial level, and 5 at the national level (excluding members of NPC or CPPCC standing bodies); for those who do not belong to the above two categories, the value is 0, while for those who have multiple political identities, the statistics are carried out according to the highest value. In this study, corporate executives (including directors, supervisors, and other executives) were disclosed, excluding independent directors.

#### 3.2.3 Other control variables

[37] provided further research on corporate growth. The following variables were controlled in this study: Business Size (*Size*), Cash Flow (*Fcf*), Market Competition (*Market*), Financial Leverage (*Lev*), Board Independence (*Bdi*), Ownership Concentration (*Fshr*), Business Age (*Age*), Intangible Assets Ratio (*Ria*), Business Risk (*Risk*), Z score (*Zscore*), and Turnover Capacity (*Turn*). S3 File provides a detailed description of these indicators.

### 3.3 Model design

To empirically test the impact of R&D innovation and political background on corporate growth, the modeling ideas of [38] were drawn from the Reduced-Form Model, which is constructed as follows:

$$Growth_{i,t+1}=\alpha_{0}+\alpha_{1}R\&D_{i,t}+\alpha_{2}Political_{i,t}+\sum_{k=1}^{n}\lambda_{i}\times Control_{it}+\varepsilon_{it}$$ (1)

In the above model, *i* represents the individual corporation, *t* represents the annual identifier, *εit* represents the random perturbation term, and *λi* represents the coefficient of the control variable. *Growth* represents corporate growth, *R&D* and *Political* represent R&D innovation and political background, respectively, and *Control* represents firm size and other control variables. In view of the time-lag effect that R&D innovation and political background may have on corporate growth, this study treats explanatory and control variables with a one-year lag in the model regression, which can also effectively reduce possible endogenous problems between variables. In this study, the coefficients of R&D innovation and political background in Model (1) were used to investigate their direct influence on corporate growth, and quantile regression was used to investigate the evolutionary trend of the marginal effect. To avoid measurement problems, such as nonlinear relations and non-stationary series in panel data, natural logarithms of all variables except dummy variables are used. This ensures that the interpretation of research results is more concise and the data distribution is closer to the normal distribution.

### 3.4 Research methods

To describe the dynamic evolution trajectory of the marginal effect of R&D innovation and political background on corporate growth based on traditional measurement methods, this study used the panel quantile regression method to estimate the parameters of Model (1). As described in [39], the ordinary least square method can only measure the marginal change in the mean value of the dependent variable caused by the marginal change in the independent variable. Therefore, regression curves corresponding to each quantile were calculated to obtain complete information on the independent variables. Based on this, the quantile regression method proposed in [40] was adopted in this study to depict the marginal effects of explanatory variables on explained variables at specific quantiles, present the distribution characteristics of different conditions of explained variables more comprehensively, and avoid the one-sided inference of real problems based on average effects. In empirical studies, the representative digits commonly used to estimate are 0.1, 0.25, 0.5, 0.75, and 0.9, and the estimated 5-digit equations are correlated. Therefore, the bootstrap method can be used to estimate the standard error of the coefficients by repeated sampling directly from the whole regression process.

Given X, assuming the quantile of the continuous random variable Y is written yτ(0<τ<1), Fy / x (y) is the cumulative distribution function of Y, yτ is a linear function, then

$$y_{\tau }=X^{T}\beta (\tau )$$ (2)

In Eq (2), β(τ) is an unknown parameter. Quantile regression calculates parameter estimates by minimizing the sum of the absolute values of the weighted errors. Therefore, the sample τ condition quantile is regarded as the optimal solution to the weighted average problem that minimizes the absolute values of the residuals; that is,

$$\overset{\wedge }{y_{\tau }}=\underset{\varepsilon }{min}\sum_{i{:y}_{i}>>\varepsilon }^{n}\tau |y_{i}-\varepsilon |+\sum_{i{:y}_{i}>>\varepsilon }^{n}\left(1-\tau )|y_{i}-\varepsilon \right|$$ (3)

By solving Eq (3), we can get the following:

$$\beta (\tau )=\arg\;\underset{\rho }{min}\;E\{\rho_{\tau }(Y-X^{T}\beta )\}$$ (4)

Considering the inherent characteristics of corporate growth rate distribution, most scholars have adopted quantile regression to study corporate growth. [41] pioneered the use of quantile regression to examine corporate growth. [42] pointed out that corporate growth rate can be positive or negative and the two states are influenced differently by different. Therefore, it is necessary to use a quantile regression method to explore the influence of each factor on the entire conditional distribution of the corporate growth rate. [12] also used the quantile regression method to study the relationship between innovation and corporate growth, and pointed out that high-growth firms, as outliers, could not be eliminated. These results indicate that innovation affects the growth rates of high-scoring firms with high scores. Based on previous studies, [33] applied quantile regression to study African firms’ high-growth problem performance.

## 4. Analysis of empirical results

### 4.1 Descriptive statistics

Table 1 presents the descriptive statistics. The average growth rate of the sample corporations was 17.6% and the standard deviation was 0.331, indicating that the growth level of corporations varied greatly. The median is 12.8%, indicating that most corporations’ growth rates are lower than the industry’s average growth rate. The mean political background of corporate executives was 0.108, indicating that only 10.8% of the sample had a political background. The mean value of R&D expenditures is 0.047, indicating that the R&D intensity of listed manufacturing corporations is 4.7%. According to the relevant description in the Oslo Manual of the Organization for Economic Cooperation and Development (OECD), the R&D investment intensity of a corporation is 1%-4%, indicating that its technological innovation capability is medium. According to this standard, China’s listed manufacturing corporates have significantly progressed in R&D innovation. The mean value of R&D personnel input is 0.177, indicating that the proportion of R&D personnel to the total number of employees is 17.7% on average, and the standard deviation is 1.734, indicating that the sample corporations have a significant difference in R&D personnel input. The observed value of this index is 4075, indicating a large number of missing values for this variable. From the perspective of the control variables, the age, Z value, scale, turnover capacity, and business risk of the corporation are generally significantly different. Cash flow, market competition, financial leverage, independence of the board of directors, ownership concentration, and the proportion of intangible assets show little difference.

**Table 1:** Descriptive statistics of main variables.

Variable	N	Mean	SD	Min	Max	P25	Median	P75
*Growth*	6079	0.183	0.336	0.606	4.874	0.015	0.132	0.282
*R&D*	6079	0.047	0.04	0	0.728	0.03	0.039	0.055
*R&D_Per*	4075	0.177	1.734	0	0.809	0.097	0.129	0.178
*political*	6079	0.108	0.311	0	1	0	0	0
*Political_lev*	6079	0.153	0.448	0	1.792	0	0	0
*Age*	6079	2.662	0.366	1.099	4.094	2.485	2.708	2.944
*Zscore*	6079	1.277	0.91	0	5.617	0.539	1.14	1.878
*Fshr*	6079	0.558	0.142	0.142	0.947	0.456	0.57	0.667
*Size*	6079	21.613	0.959	17.654	26.298	20.9	21.509	22.194
*Bdi*	6079	0.376	0.054	0.2	0.75	0.333	0.333	0.429
*Turn*	6079	0.605	0.388	0.022	7.609	0.391	0.536	0.732
*Ria*	6079	0.046	0.036	0	0.589	0.025	0.039	0.057
*Market*	6079	0.089	0.098	0.001	0.801	0.031	0.056	0.107
*Fcf*	6079	0.168	0.133	0	0.837	0.074	0.127	0.223
*Risk*	6079	1.11	0.311	0.609	3.643	0.938	1.087	1.25
*Lev*	6079	0.335	0.174	0.008	1.279	0.198	0.318	0.456

This study also examines changing trends in R&D innovation and political backgrounds among the sample firms by year. Table 2 and S1 Fig show that the intensity of the sample corporates’ R&D innovation input during 2012-2019 shows an upward trend, and the proportion of R&D innovation input in corporate revenue gradually increased. However, the intensity of political background shows an apparent downward trend. The input of the sample corporations to build political backgrounds decreases annually, and the gap between them and the intensity of R&D innovation inputs increases annually.

**Table 2:** Annual changes of R&D innovation and political background.

Variables	2012	2013	2014	2015	2016	2017	2018	2019
*R&D (mean)*	0.0434	0.0457	0.0446	0.0464	0.0486	0.0482	0.0511			0.0560
*Political_lev (average)*	1.0304	0.9714	0.9703	0.6811	0.6704	0.7782	0.6656		0.5652

### 4.2 Test of correlation coefficients of major variables

Table 3 shows the Pearson correlation coefficient matrix of the main variables, all of which are lower than 0.5, indicating that there is no serious multicollinearity problem between the variables. *R&D* has a significantly positive correlation with *Growth*, whereas *Political* has a negative but insignificant correlation with *Growth*. However, Pearson’s correlation coefficient only reflects the degree of correlation between variables and does not reflect the causal relationship between variables. Therefore, regression analysis should be conducted on the explanatory variables while controlling for relevant factors to accurately determine the degree of influence, direction of the relationship, and significance level between the dependent and explanatory variables.

**Table 3:** Correlation coefficients between the main variables.

	Growth	R&D	political	Age	Zscore	Fshr	Size
*Growth*	1.000							
*R&D*	0.077***	1.000						
*political*	0.010	0.071***	1.000					
*Age*	0.081***	0.043***	0.025*	1.000				
*Zscore*	0.008	0.054***	0.004	0.009	1.000			
*Fshr*	0.035***	0.039***	0.018	0.113***	0.018	1.000		
*Size*	0.052***	0.174***	0.020	0.082***	0.088***	0.140***	1.000	
*Bdi*	0.030**	0.075***	0.016	0.021	0.011	0.080***	0.082***	
*Turn*	0.092***	0.282***	0.054***	0.031**	0.024*	0.052***	0.076***	
*Ria*	0.031**	0.016	0.039***	0.013	0.010	0.012	0.022*	
*Market*	0.001	0.151***	0.007	0.061***	0.012	0.073***	0.075***	
*Fcf*	0.002	0.181***	0.012	0.089***	0.047***	0.166***	0.285***	
*Risk*	0.003	0.053***	0.032**	0.009	0.014	0.069***	0.146***	
*Lev*	0.005	0.206***	0.009	0.044***	0.083***	0.176***	0.497***	
	Bdi	Turn	Ria	Market	Fcf	Risk	Lev	
*Bdi*	1.000							
*Turn*	0.049***	1.000						
*Ria*	0.007	0.047***	1.000					
*Market*	0.025*	0.104**	0.036***	1.000				
*Fcf*	0.006	0.082***	0.128***	0.175***	1.000			
*Risk*	0.001	0.011	0.031**	0.091***	0.030**	1.000		
*Lev*	0.032**	0.124***	0.041***	0.185***	0.427***	0.032**	1.000	

Note

***, **, and * indicates that the parameter estimates are significant at the 1%, 5%, and 10% levels, respectively.

### 4.3 Analysis of regression results

To comprehensively reflect the group differences in corporations with different growth rates, this study selected five representative groups: a declining corporate group (Quan10), a low-speed growth corporate group (Quan25), a medium-speed growth corporate group (Quan50), a medium-to-high-speed growth corporate group (Quan75), and a high-speed growth corporate group (Quan90). Table 4 presents the main regression results of the model. The leftmost column shows the regression results of the panel fixed effects model and the five columns on the right show the results of the panel quantile model regression. From left to right, the estimated equations are 0.10, 0.25, 0.50, 0.75, and 0.90. The results are based primarily on the regression results of the panel quantile model. Panel fixed-effects model regression results were used for comparison and reference.

**Table 4:** Hierarchical effects of R&D innovation and political association on corporate growth.

Variables	Fixed effects model	Panel quantile regression models				
		Quan10	Quan25	Quan50	Quan75	Quan90
*R&D*	0.536*** (4.45)	0.120*** (13.97)	0.312*** (53.85)	0.584*** (189.92)	0.738*** (61.06)	1.288*** (71.87)
*political*	5.33 e-05 (0.00)	-0.016*** (-4.01)	-0.008*** (-4.74)	0.013*** (15.23)	0.026*** (17.54)	0.050*** (15.42)
*Age*	0.069*** (5.31)	0.013*** (4.35)	-0.018*** (-23.05)	-0.034*** (-64.75)	-0.098*** (-66.68)	-0.017*** (-8.25)
*Zscore*	0.005 (0.96)	-0.003*** (-5.33)	-0.003*** (-7.67)	-0.002*** (-6.09)	0.003*** (6.17)	-0.050*** (-41.99)
*Fshr*	0.109*** (3.39)	0.159*** (81.46)	0.118*** (35.29)	0.058*** (38.07)	0.021*** (8.89)	0.361*** (51.61)
*Size*	0.022*** (4.00)	-0.003 (-1.61)	0.004*** (13.15)	-0.006*** (-27.95)	-0.017*** (-30.03)	-0.088*** (-113.27)
*Bdi*	0.080 (0.97)	0.024 (1.13)	-0.046*** (-9.44)	0.052*** (6.54)	0.223*** (24.69)	-0.095*** (-5.47)
*Turn*	0.063*** (5.32)	-0.009** (-2.04)	-0.006*** (-7.95)	-0.029*** (-38.81)	-0.104*** (-67.19)	-0.685*** (-91.85)
*Ria*	0.154 (1.25)	0.219*** (9.53)	0.118*** (8.69)	0.062*** (8.12)	-0.515*** (-34.60)	0.838*** (41.69)
*Market*	0.025 (0.53)	0.132*** (30.91)	0.090*** (20.95)	0.011*** (3.42)	-0.135*** (-20.51)	-0.189*** (-29.13)
*Fcf*	0.059 (1.52)	-0.081*** (-16.24)	-0.003 (-1.53)	-0.005** (-2.28)	-0.047*** (-10.06)	-0.137*** (-19.57)
*Risk*	0.030** (1.97)	0.024*** (7.00)	0.023*** (28.84)	0.012*** (12.65)	-0.029*** (-14.29)	-0.024*** (-10.93)
*Lev*	0.096*** (3.05)	-0.039*** (-5.39)	0.012*** (7.61)	0.056*** (9.02)	0.082*** (33.07)	0.298*** (61.59)
Year	Control	Control	Control	Control	Control	Control
N	6079	6079	6079	6079	6079	6079

Note

***, **, and * indicate that the parameter estimates are significant at the 1%, 5%, and 10% levels, respectively, and the internal values of () are the t-statistic (fixed effects model) or Z-statistic (panel quantile model). The labeling error of the variable coefficient under the panel quantile model was obtained using the bootstrap method with 1000 times.

First, through a panel fixed-effects analysis of the sample data, the elasticity coefficient of R&D innovation on corporate growth was found to be significantly positive. For every 1% increase in R&D innovation intensity, the corporate growth rate increased by 0.536%, indicating that R&D innovation significantly promotes corporate growth at the average level. The coefficient of elasticity of political background on corporate growth is 5.33E-05, which is insignificant and indicates that political background has no significant influence on corporate growth at the average level. Second, according to the estimated results of the panel quantile regression model, the following findings can be summarized: R&D innovation positively impacts corporate growth, which is consistent with many findings in the existing literature. From the perspective of hierarchical differences, the positive effect of R&D innovation varies significantly across the quantiles of the conditional distribution of corporate growth rate. Columns (2)–(6) of Table 4 show the regression results for 0.10, 0.25, 0.50, 0.75, and 0.90. The estimated coefficients of R&D innovation are 0.120, 0.312, 0.584, 0.738, and 1.288, all of which are significant at the 1% level. These coefficients show the marginal effect of changes in R&D innovation on each corporate growth rate. It is not difficult to find that the positive impact of changes in R&D innovation on the growth rate increases with an increase in the quantile, and the R&D innovation coefficient at 0.90 points is more than 10 times higher than that at 0.10 points.

Therefore, the promotional effect of R&D innovation is evident in firms with high growth rates. When the firm is in a recession due to business failure (growth in the 0.1 digits), the effect of R&D innovation on firm growth is less obvious. Therefore, panel quantile regressions can provide more comprehensive information than panel fixed-effects regressions. This is because the panel fixed effect (0.536) overestimates the promoting effect of R&D innovation on corporations during recession periods and underestimates the promoting effect of R&D innovation on corporations during rapid growth periods. These results also indicate that corporate growth does not follow Gibrat’s law. In addition, these results suggest that R&D innovation has a protective effect on corporate growth even in the face of a firm recession. The influence of political backgrounds on corporate growth differs significantly across locations. At the low quantile of 0.10 and 0.25, the coefficient of political background is significantly negative, indicating that political background hinders corporate growth for firms with a low growth rate. The coefficient of political background changes from negative to positive at 0.50, and increases with an increase in the corporate growth rate from 0.013 at 0.50 to 0.050 at 0.90. Political backgrounds can significantly promote the growth of high-growth corporations. The above distributions tend to "cancel each other out" in the mean reversion, which is one reason why the test of the mean reversion results is insignificant. These results also suggest that the political background accelerates the contraction of recessionary firms and the expansion of high-growth firms.

In summary, the empirical results show that an increase in R&D innovation investment intensity promotes corporate growth, and that R&D innovation has a heterogeneous effect. R&D innovation has a more substantial promoting effect on corporations’ high growth rates (corresponding to the high score locus) than on corporations’ low growth rates (corresponding to the low score locus). The political background has heterogeneous effects on firm growth. Political background promotes the growth of firms with high growth rates, but inhibits that of firms with low growth rates. This result is similar to the conclusion of [43] on the "winner selection strategy" of government subsidies. Therefore, H1, H2, and H3 were assumed valid.

Table 4 also shows the influence of the control variables on corporate growth. Through a panel fixed-effects analysis of the sample data, we find that corporate age, size, turnover capacity, and business risk significantly and negatively impact corporate growth. By contrast, equity concentration and financial leverage have a significant positive impact on corporate growth. Z-value, board independence, proportion of intangible assets, market competition, and cash flow do not significantly impact corporate growth. However, the panel quantile regression model provides a more detailed and different estimation result: the regression coefficient of firm age on the growth of recessionary firms is 0.013 and is significant at the 1% significance level. However, for the growth of firms at 0.25, 0.50, 0.75, and 0.90, the regression coefficient is negative and significant at the 1% significance level, indicating that the growth in firm age can alleviate the further contraction of recessionary firms. This is because mature corporations have more robust learning and absorption abilities, richer market experience and management knowledge, and a greater ability to deal with uncertainty, which helps them cope with the current recession more calmly based on their previous growth experience. Mature corporations can simultaneously gain wider market reputation and status over time. This helps build strong relationships with customers, suppliers, and partners, so that mature corporations can obtain more external support to overcome poor management difficulties in recessions. Additionally, young firms are often less productive than established firms when they enter the market and it takes them time to adapt to the operating environment and improve their internal capabilities [44]. Thus, young companies tend to underperform when facing a performance downturn. Some scholars note that corporate survival rates increase with age, and those most likely to quit the market are increasingly smaller and younger [18]. However, mature businesses are also affected by headwinds that prevent them from achieving higher growth rates. Existing studies indicate that organizational inertia limits a corporation’s ability to change, and mature corporations are trapped in inertia, which hinders their learning effects. Therefore, the regression coefficients of firm age on corporate growth are 0.25, 0.50, 0.75 and 0.90 quantiles are all negative.

The influence of firm size on corporate growth exhibits significant hierarchical differences. Except for the positive coefficient at 0.25, firm size affects corporate growth at each quantile, and the negative effect is significantly enhanced with an increase in the quantile. The restrictive effect of firm size is positively correlated with corporate growth rate. When the corporation is in recession due to poor management (growth in the 0.10 digit), the restrictive effect of size on the growth of the corporation is not obvious, but when the corporation is in the growth stage of the high score (such as 0.75 and 0.90), the restrictive effect of size is very obvious. Therefore, panel quantile regression can provide more comprehensive information than panel fixed-effects regression. This is because the panel fixed effect (0.536) overestimates the promoting effect of R&D innovation on corporations in a recession period but underestimates it in a rapid growth period. According to Gibrat’s law, the growth of corporations is random and unpredictable in advance; small corporations will encounter a small growth impact, and large corporations will encounter a significant growth impact, so the growth rate of corporations has nothing to do with the size of corporations. In a certain period, small and large corporations may achieve a certain speed of growth, and our conclusions do not support Gibrat’s law.

The Z-value coefficient is only significantly positive at 0.75 quantile and significantly negative at the level of the remaining quantiles, indicating that an increase in the Z-value is unfavorable to the growth of most corporations. The coefficient of ownership concentration is significantly positive at all quantile levels, and its influence on the two ends of the conditional distribution of the corporate growth rate is greater than its influence on the middle part, indicating that ownership concentration has a more significant impact on high-speed growth or declining corporations. The regression coefficients of board independence at the different quantile levels differ significantly. It has the largest negative impact on the growth of high-speed growth corporations, a significant positive impact on the growth of medium- and medium-high-speed growth corporations, and no significant impact on declining corporations. The coefficient of turnover capacity is significantly negative at each quantile level, and the negative influence becomes increasingly significant with an increase in the quantile. The coefficient of the intangible assets ratio is significantly negative only at the 75% quantile level and significantly positive at the other quantile levels, indicating that an increase in the intangible assets ratio is conducive to the growth of most corporates. The influence of market competition on the two ends of the conditional distribution of corporate growth rate is more significant than that on the middle part and it is significantly positive at the 10%, 25%, and 50% quantiles. Additionally, the positive effect gradually weakens with an increase in quantile, whereas it is significantly negative at the 75% and 90% quantile levels. Market competition had the most substantial inhibitory effect on high-speed firms’ growth. The cash flow coefficient is negative and significant at each quantile level except at the 25% quantile level. At the same time, the influence of cash flow on both ends of the conditional distribution of corporate growth rate is more significant than in the middle, indicating that cash flow is more critical when corporations grow at high speeds and during recessions. At the low and medium quantiles (0.10, 0.25, and 0.50), the coefficient of business risk is significantly positive, while at the medium-high and high quantiles (0.75, 0.90), the coefficient of business risk is significantly negative, indicating that business risk hinders the rapid growth of corporations, but plays a specific role in mitigating their recession. The coefficient of financial leverage at the 10% quantile level is significantly negative, but in the rest of the quantile levels is significantly positive, and with the rise of the quantiles, the coefficient of financial leverage increases, indicating that financial leverage will further aggravate the contraction of recession-type corporations. However, the boosting effect of financial leverage was pronounced for corporations in the growth stage.

### 4.4 Robustness test

A test was conducted to verify the robustness of the results. The scientific robustness test strongly supported the empirical results described above.

In this study, the ratio of R&D staff was used as a surrogate variable for R&D innovation intensity and the level of political background was used as a surrogate variable for political background. The estimated analysis of the panel fixed-effects model and the panel quantile regression model was repeated. Table 5 presents the regression results. The results of the robustness test were based mainly on the regression results of the panel quantile model, and the regression results of the panel fixed-effects model were used for comparison and reference. First, through a panel fixed effects analysis of the sample data, it was found that R&D innovation and political associations both have a positive impact on corporate growth, but they are not significant at the 10% significance level. Second, according to the estimated results of the panel quantile regression model, we find that the coefficient of R&D innovation is significantly positive at each quantile level and that the positive effect of R&D innovation becomes stronger with the growth of the quantile. The R&D innovation coefficient at 0.90 is four times that at 0.10, again proving the heterogeneous impact of R&D innovation on corporate growth. Compared with corporations with a low growth rate (corresponding to the low quantile), R&D innovation has a more substantial promoting effect on the growth of corporations with a high growth rate (corresponding to the locus of the high score). The political background level has a significant negative effect on corporate growth in the low quantile (0.10 and 0.25) and a significant positive effect on corporate growth in the middle and high quantiles (0.50, 0.75, and 0.90). Moreover, the political background level has the strongest promotional effect on high-growth corporations and the greatest inhibitory effect on declining corporations. The regression results confirm that political backgrounds have heterogeneous effects on firm growth. Political background promotes the growth of firms with high growth rates and inhibits the growth of firms with low growth rates. The regression results for the remaining control variables are consistent with those in Table 4 and are not presented individually.

**Table 5:** Changes the robustness test for the core explanatory variables.

Variables	Fixed effects model	Panel quantile regression models				
		Quan10	Quan25	Quan50	Quan75	Quan90
*R&D_Per*	0.003 (0.88)	0.001*** (13.97)	0.001*** (53.93)	0.002*** (171.16)	0.003*** (217.59)	0.004*** (73.91)
*Political_lev*	0.003 (0.24)	-0.005** (-2.09)	-0.001*** (-9.12)	0.014*** (11.18)	0.022*** (30.08)	0.032*** (7.04)
*Age*	-0.073*** (-4.37)	-0.052*** (-12.86)	-0.024*** (-18.23)	-0.069*** (-18.01)	-0.141*** (-106.18)	-0.159*** (-31.22)
*Zscore*	0.002 (0.30)	0.008*** (6.18)	-0.008*** (-20.09)	-0.004*** (-7.53)	-0.002*** (-11.45)	0.006*** (3.64)
*Fshr*	0.199*** (5.17)	0.249*** (16.84)	0.115*** (56.75)	0.111*** (16.65)	0.039*** (11.37)	0.104*** (21.16)
*Size*	-0.016** (-2.56)	0.017*** (22.68)	0.005*** (9.650)	-0.007*** (-12.86)	-0.016*** (-57.36)	-0.067*** (-29.42)
*Bdi*	0.133 (1.38)	-0.080** (-2.35)	-0.006 (-0.57)	0.063*** (7.44)	0.171*** (19.23)	0.375*** (14.50)
*Turn*	-0.073*** (-5.02)	0.007** (2.19)	-0.039*** (-20.770)	-0.059*** (-20.68)	-0.082*** (-203.20)	-0.151*** (-21.51)
*Ria*	0.133 (0.94)	0.336*** (7.45)	0.273*** (24.99)	0.131*** (18.46)	0.182*** (15.41)	-0.058** (-2.17)
*Market*	-0.068 (-1.28)	0.006 (0.64)	-0.073*** (-7.94)	-0.024*** (-5.12)	-0.107*** (-54.23)	-0.382*** (-34.49)
*Fcf*	-0.072 (-1.46)	-0.105*** (-8.31)	0.025*** (9.31)	0.021*** (5.39)	-0.004 (-1.41)	-0.073*** (-12.53)
*Risk*	-0.011 (-0.69)	0.072*** (50.68)	0.014*** (9.78)	0.010*** (8.91)	0.004*** (3.50)	-0.067*** (-9.46)
*Lev*	0.100*** (2.69)	-0.133*** (-20.57)	-0.022*** (-11.87)	0.084*** (14.65)	0.126*** (27.59)	0.374*** (33.45)
*Year*	Control	Control	Control	Control	Control	Control
*N*	4075	4075	4075	4075	4075	4075

Note

***, **, and * indicate that the parameter estimates are significant at the 1%, 5%, and 10% levels, respectively, and the internal values of () are the t-statistic (fixed effects model) or Z-statistic (panel quantile model). The labeling error of the variable coefficient under the panel quantile model was obtained using the bootstrap method with 1000 times.

### 4.5 Further analysis

#### 4.5.1 Examination of relative importance

Corporations find it difficult to achieve sustainable development solely through internal resource allocation. Corporate growth requires the acquisition of external resources. However, owing to constraints such as transaction and financial distress costs, corporations cannot obtain all the necessary resources. With limited resources, corporations can optimize the allocation of resources and invest more in activities that maximize their value. R&D innovation or the construction of political background requires corporations to pay a specific cost. Corporations with limited resources, have to choose between R&D innovation and political background. In these two instances, it is necessary to clarify the strength and weakness of the promoting effect on corporate growth to make reasonable resource allocation decisions based on their growth situation.

Based on this analysis, we use a standardized regression to examine the relative importance of R&D innovation and political background in corporate growth. First, according to Table 4, under the panel fixed-effects model, the R&D innovation coefficient is significantly positive, while the political background coefficient is also positive but not significant. Under the panel quantile regression model, for corporations whose growth rates are distributed in the 0.10 and 0.25 quantile, the coefficient of R&D innovation is significantly positive, whereas the coefficient of political background is significantly harmful. Therefore, section 4.5.1 does not investigate the relative importance of the fixed effects model and corporate samples for 0.10 the 0.25 quantile. Table 6 shows that in the standardized regression results, the coefficients of R&D innovation at each quantile are 0.073, 0.098, and 0.146. In contrast, the coefficients of political background correspond to 0.006, 0.002, and 0.001, indicating that after the elimination of the dimension, the contribution of unit R&D innovation is more significant than that of unit political background; that is, relative to political background, R&D innovation is more critical to corporate growth. An outstanding characteristic of Chinese corporations is that there is no obvious linear relationship between political background and firm age. Some young companies may receive significant policy support, whereas mature companies may have a weak political background because of their mature business models. Therefore, we examined the moderating effect of firm age on R&D innovation and corporate growth.

**Table 6:** Test results of relative importance.

Variables	Panel quantile regression model		
	Quan50	Quan75	Quan90
*R&D*	0.073*** (102.54)	0.098*** (25.63)	0.146*** (63.43)
*political*	0.006*** (24.59)	0.002*** (8.02)	0.001 (1.00)
*Age*	0.016*** (23.68)	0.032***(12.59)	0.079***(160.97)
*Zscore*	0.003*** (15.05)	0.000 (0.22)	0.005*** (4.82)
*Fshr*	0.009*** (21.39)	0.015*** (6.12)	0.006*** (5.42)
*Size*	0.011***(25.76)	0.026***(9.04)	0.092***(41.29)
*Bdi*	0.002* (1.91)	0.007** (2.19)	0.029*** (19.15)
*Turn*	0.050***(93.45)	0.191***(8.33)	0.354***(15.50)
*Ria*	0.002*** (5.49)	0.004**(1.99)	0.003 (0.66)
*Market*	0.001*** (4.12)	0.025***(8.14)	0.051***(29.71)
*Fcf*	0.002***(5.05)	0.025***(5.75)	0.026***(11.48)
*Risk*	0.007*** (4.89)	0.007***(2.80)	0.035***(19.28)
*Lev*	0.020*** (48.66)	0.023***(27.18)	0.088*** (34.46)
*Year*	Control	Control	Control
*N*	6079	6079	6079

Note

***, **, and * indicate that the parameter estimates are significant at the 1%, 5%, and 10% levels, respectively, and the internal values of () are the t-statistic (fixed effects model) or Z-statistic (panel quantile model). The labeling error of the variable coefficient under the panel quantile model was obtained using the bootstrap method with 1000 times.

#### 4.5.2 Moderating effect of firm age on the relationship between R&D innovation and corporate growth

Many studies show that young firms have higher growth rate [44, 45]. Compared with mature corporations, young corporations introduce innovations more frequently to survive in the market [46–48], are more inclined toward radical innovation [37, 49], and have higher R&D intensity [50]. Mature corporations are more inclined to conduct process innovations and incremental improvements in existing technologies. Simultaneously, young companies can discover new markets or technological opportunities from a "fresh understanding" of the industry status quo without being hampered by organizational inertia or worrying about whether new products or services will encroach into the existing market share. In addition, the flexibility of young corporations’ organizational structure makes them more inclined toward radical innovation, whereas mature corporations constrained by existing resources and customer bases tend to choose more conservative innovation [51]. However, in the initial stage of market entry, young corporations lack a sound operating system. Therefore, they must quickly build an operating system and cultivate internal R&D capabilities. Consequently, young corporations may initially lack the ability to benefit from R&D investments [52]. Empirical evidence also shows that young firms often have lower productivity levels than incumbent firms when they enter the market and that it takes time for young firms to adapt to the operating environment and improve their internal capabilities [44]. [37] used quantile regression to find that young corporations are more inclined toward radical and riskier R&D projects, thus achieving higher growth rates. However, young corporations have a higher probability of innovation failure given their lack of experience. [53] show that business survival increases with age and that firms that are most likely to exit the market are smaller and younger. This analysis shows that the relationship between young corporations’ R&D innovation activities and growth needs to be clarified through further research.

Relevant studies have highlighted the existence of learning effects. Mature enterprises have relatively more robust learning and absorption capabilities, which help them achieve better performance and growth through R&D innovation based on previous knowledge accumulation [54, 55]. Over time, corporations can accumulate more resources, management knowledge, and the ability to deal with uncertainty and gain a broader market reputation and position, which helps them connect with customers, suppliers, and partners [37]. There is also evidence that business age positively impacts organizational excellence [56], new product development [57], and the success of R&D projects [54]. [12] believe that with the passage of time, corporations would gain valuable experience, ability, and understanding, and communication and cooperation between R&D personnel would become more efficient. Therefore, R&D activities should not be interrupted and long-term R&D activities are the most effective [18]. [58] noted that a corporation’s past R&D activities can significantly improve its current R&D efficiency. [59] describe R&D output as an increasing return on knowledge generation. Corporations can use their existing knowledge stock to create new knowledge and knowledge accumulation can significantly improve R&D efficiency. The more a corporation learns in the past, the higher its success rate in achieving future innovation-driven growth. [60] found that high-intensity R&D expenditure substantially affects mature corporations. The analysis showed that R&D innovation plays a positive role in mature corporations. However, mature firms are affected by several adverse factors preventing them from translating their R&D investments into higher growth rates. Existing studies point out that organizational inertia limits a corporation’s ability to change; mature corporations will be trapped by their own inertia, thus hindering their learning effect, and when the innovation activities of mature corporations based on previous experience do not match the external technological environment, the innovation effect will be significantly reduced. Further research is required to clarify the relationship between the R&D innovation activities of mature corporations and growth.

The most intuitive conclusion from this analysis is that the effect of R&D innovation on corporate growth is primarily influenced by a firm age [37, 46, 47, 61]. Another prominent feature of Chinese corporations is that their average R&D intensity is lower than mature corporations. This phenomenon contrasts the statistical results for high-income countries. Microdata from Italy, the UK, and Spain show that young corporations tend to have higher R&D intensity than mature corporations [37, 47, 61]. Given that the existing research on the relationship between corporate age and innovation and corporate growth is not clear, and that the outstanding characteristics of Chinese corporates also need further empirical analysis to draw a clear conclusion, this study based on the sample corporates division (young corporates: age ≤10 years. Mature corporations: >10 years) to analyze the effect of corporate age on the relationship between R&D innovation and corporate growth.

The following regression model was constructed to test the moderating effect of firm age:

$$\begin{array}{l} Growth_{i,t+1}=\varphi_{0}+\varphi_{1}R\&D_{i,t}+\varphi_{2}Political_{i,t}+\varphi_{3}Young_{i,t}+\varphi_{4}R\&D_{i,t}\times Young_{i,t}\\ \;+\sum_{k=1}^{n}\lambda_{i}\times Control_{it}+\varepsilon_{it} \end{array}$$ (5)

where *i* represents an individual business, *t* represents the annual identifier, *εit* represents the random perturbation term, and *λi* represents the coefficient of the control variable. *Growth* represents corporate growth; *R&D* and *Political* represent R&D innovation and political background, respectively; *Young* represents whether the corporation is young; if the corporation is less than 10 years old, the dummy variable is equal to 1; otherwise, it is equal to 0; *Control* represents corporate size and other control variables.

According to Table 7, the panel fixed effect regression shows that the elasticity coefficient of the *Young*’s variable is significantly positive at the 1% level, and the interaction term *Young×R&D* coefficient is negative and insignificant, indicating that, on average, young firms have a higher growth rate. However, the effect of young firms’ R&D innovation activities on corporate growth remains unclear. As can be seen from the panel quantile regression results, as the growth rate of corporates moves from the low end of conditional distribution to the high end, the elastic coefficient of *Young* increases gradually, from 0.007 at the 0.10 points to 0.123 at the 0.90 points, and the elastic coefficient at each point is significant at 1% level. This shows that young corporations can achieve a higher growth rate and, during a corporate recession, they can take more flexible measures to deal with the predicament of poor management and restrain further contractions. The coefficient of the interaction term *Young×R&D* varies with the different distribution locations of the growth rate conditions, showing a significant inhibitory effect at the medium- and low-quantiles (0.50 and below) and a significant promoting effect at the medium-high- and high-quantiles (0.75 and 0.90). The coefficient is the smallest at the 0.10 points (-0.326). The most significant coefficient (0.442) for 0.75 quantiles. Thus, young corporations can achieve higher growth (i.e., higher upside returns) in the upper quantile of the conditional distribution of the corporate growth rate through R&D innovation. However, they face a greater risk of loss (i.e., more significant downside losses) if innovation fails, whereas mature corporations are less likely to conduct R&D innovation activities. Mature firms have more balanced returns in the conditional growth rate distribution. Therefore, the role of R&D innovation in young firms is stronger.

**Table 7:** Moderating effects of the corporate age.

Variables	Fixed effects model	Panel quantile regression models				
		Quan10	Quan25	Quan50	Quan75	Quan90
*R&D*	0.605*** (4.82)	0.177*** (12.09)	0.417*** (67.26)	0.639*** (157.57)	0.779*** (100.24)	1.353*** (58.98)
*political*	0.007 (0.02)	0.016*** (9.58)	0.014*** (13.41)	0.011*** (13.23)	-0.043*** (-13.85)	0.001 (0.81)
*Young*	0.053*** (4.49)	0.007*** (4.71)	0.018*** (22.67)	0.028*** (66.12)	0.047*** (33.11)	0.123*** (57.81)
*Young by R&D*	0.340 (1.42)	-0.326*** (-13.24)	-0.242*** (-8.33)	-0.060*** (-5.41)	0.442*** (10.45)	0.144*** (8.34)
*Zscore*	0.004 (0.85)	-0.003*** (-6.45)	-0.004*** (-12.07)	-0.003*** (-17.58)	0.001* (1.92)	0.008*** (12.22)
*Fshr*	0.114*** (3.55)	0.097*** (27.39)	0.116*** (45.15)	0.065*** (46.75)	0.146*** (28.55)	0.147*** (24.96)
*Size*	-0.023*** (-4.13)	0.009*** (38.91)	0.003*** (18.04)	-0.006*** (-21.89)	-0.012*** (-30.38)	-0.017*** (-13.55)
*Bdi*	0.078 (0.95)	-0.141*** (-26.12)	-0.037*** (-12.46)	0.017*** (5.00)	0.464*** (24.97)	0.146*** (14.06)
*Turn*	-0.063*** (-5.34)	0.004** (2.47)	-0.002** (-2.51)	-0.024*** (-82.73)	-0.119*** (-35.66)	-0.223*** (-151.04)
*Ria*	0.158 (1.29)	0.464*** (17.32)	0.097*** (15.60)	0.073*** (18.64)	-0.163*** (-18.15)	-0.612*** (-13.59)
*Market*	-0.033 (-0.71)	0.084*** (18.43)	0.082*** (82.70)	0.047*** (27.44)	-0.141*** (-31.19)	-0.238*** (-25.97)
*Fcf*	-0.057 (-1.46)	-0.058*** (-16.39)	-0.042*** (-26.05)	0.022*** (20.99)	-0.082*** (-26.05)	-0.243*** (-35.55)
*Risk*	-0.029* (-1.92)	0.051*** (82.24)	0.021*** (15.23)	0.007*** (8.17)	0.027*** (14.15)	-0.080*** (-35.95)
*Lev*	0.102*** (3.22)	-0.123*** (-19.26)	-0.001 (-0.63)	0.080*** (82.39)	0.136*** (60.57)	0.330*** (116.11)
*Year*	Control	Control	Control	Control	Control	Control
*N*	6079	6079	6079	6079	6079	6079

Note

***, **, and * indicate that the parameter estimates are significant at the 1%, 5%, and 10% levels, respectively, and the internal values of () are the t-statistic (fixed effects model) or Z-statistic (panel quantile model). The labeling error of the variable coefficient under the panel quantile model was obtained using the bootstrap method with 1000 times.

## 5. Discussion

Through the above theoretical analyses and empirical tests, two questions were answered: (1) What are the different effects of R&D innovation on corporate growth at different growth levels? (2) What are the effects of political backgrounds on corporate growth at different levels? Existing studies have drawn divergent conclusions regarding the combined impact of R&D innovation and political backgrounds on corporate growth. Their research conclusions are positive, negative or U-shaped, or inverted U-shaped without detailed analysis based on corporate growth features. Our analysis results presented in section 4 provides a helpful supplement to address this issue.

Our analysis results show that R&D innovation has a significant promoting effect on corporate growth, consistent with the conclusions of many existing studies. However, from the perspective of hierarchical differences, the promoting effect of R&D innovation on corporate growth varies significantly in the different quantiles of the distribution of growth rate conditions. With an increase in the quantile, the growth rate increases from very small (negative) to very large (positive), and the positive effect of R&D innovation is significantly enhanced. The R&D innovation coefficient at the 0.90 quantiles (high-growth corporations) is more than 10 times higher than that at 0.10 quantiles (declining corporates). In other words, the higher the growth rate, the greater the promotional effect of R&D innovation. Therefore, the effect of R&D innovation on corporate growth must be combined with corporate growth characteristics, ignoring the growth differences between corporations. It is insufficient to analyze the role of R&D innovation broadly.

Corporate managers should focus on innovation and development, especially for high-speed growth corporations, and increase the intensity of investment in R&D innovation to avoid being bound by growth inertia and falling into the "growth trap." Enterprises with poor management and signs of recession should consider their own resource status, maintain a prudent decision-making attitude, reasonably plan their resources invested in R&D innovation, and adopt more strategies of imitation innovation or subsequent innovation. This will avoid significant risk impact on enterprises due to radical innovation failure. The influence of political background on corporate growth also differs significantly at different loci. For low-growth corporations, the political background hinders growth, whereas for high-growth corporations, it can significantly promote growth. This shows that the political background accelerates the contraction of recessionary corporations and the expansion of high-speed growth corporations. Recession-type corporations should try to avoid investing too many corporate resources in the construction of a political background, and further clarify the strategic view that relying only on R&D innovation can timeously eliminate a recession. Corporations with good growth momentum can consider putting part of their resources into constructing political background and better promoting their growth utilizing the government’s “helping hand.”

According to the different analysis results, for corporations at any growth stage, the contribution of a unit’s R&D innovation is greater than that of a unit’s political association; that is, relative to political associations, R&D innovation is more critical to corporate growth. With limited resources, rational entrepreneurs choose to invest more in activities that create maximum value for corporations. Thus, corporations should invest more resources in R&D innovation. To ensure a sufficient supply of the resources required for R&D projects, some resources can be considered for investment in the construction of political backgrounds. However, political background has a tremendous negative impact on recession-type corporations. Therefore, the construction of the political background should be carefully selected.

Age primarily affects the impact of R&D innovation on corporate growth. Young firms can enjoy higher growth rates, and when a downturn occurs, they can take more flexible measures to deal with poor management, thus preventing further contraction. Additionally, R&D innovation can achieve higher growth (i.e., higher upside returns) in the upper quantile of the conditional distribution of the corporate growth rate for young corporations. However, if innovation fails, they face a greater risk of loss (i.e., more significant downside losses), whereas, for mature corporations, the risk of R&D innovation activities is lower. Mature firms have more balanced returns in their conditional growth rate distributions. Thus, R&D innovation plays a crucial role in young firms. Compared with mature corporations, young corporations introduce innovation more frequently, are more inclined toward radical innovation, and have a higher R&D intensity to survive in the market. However, in the initial stages of entering the market, young corporations lack a sound operational system, resulting in a lack of internal capacity to benefit from R&D investment. Therefore, young corporations must formulate appropriate R&D investment intensities, based on their R&D and management abilities. They should not be divorced from their own resources nor blindly conduct R&D activities to avoid the irreparable losses caused by radical innovation. Mature corporations are more inclined to engage in process innovation and progressive improvements in existing technologies. Constrained by existing resources and customer bases, they choose more conservative innovations. Additionally, organizational inertia limits a corporation’s ability to change. Mature corporations will be trapped by their inertia, thus hindering their learning effect. When the innovation activities of mature corporations based on previous experience do not match the external technology environment, the innovation effect will be significantly reduced. Therefore, mature corporations should avoid the negative impact of organizational inertia on their further growth, not only limited to relatively conservative innovation links, but should also further increase investment in R&D projects with higher technology content and broader market prospects to stabilize their competitive market position.

## 6. Limitations and future research directions

Through the above empirical studies, this study verifies the heterogeneous relationship between R&D innovation, political background, and corporate growth; however, some limitations still provide directions for future research.

First, the sample selected for this study has several limitations. The data on Shanghai and Shenzhen A-share private manufacturing listed companies used in this study have certain advantages in terms of the number of samples and types of corporations. However, the empirical results are not necessarily applicable to other industries given it represents the influence of a single manufacturing industry. The effects of services and other industries must be further verified.

Second, we use the proportion of R&D expenditure in central business income as the intensity of R&D investment to measure corporations’ R&D innovation behavior. Future studies could measure R&D innovation results by distinguishing the proportion of investments that form novel patents from those that do not.

Finally, the forms of political associations were diverse. For example, relatives and friends of corporate managers have a political background or hold positions in relevant government departments, or corporate managers are members of industry associations, trade unions, and so on, and have cooperative relations with government departments. Subsequent studies could focus on whether different forms of political association have different impacts on the development of corporations.

## 7. Conclusion

Although many studies have examined the impact of R&D innovation and political backgrounds on corporate growth, our study is the first to combine them within the same framework and comprehensively discuss their impacts on corporate growth. This study considers the influence of different growth rates and corporate age. We find that R&D innovation and political background directly affect corporate growth and have heterogeneous effects at different corporate growth levels. That is, R&D innovation has a strong promoting effect on firms with a high growth rate, while political background inhibits the growth of corporations with a low growth rate but promotes the growth of corporations with a high growth rate. Compared with the political background, R&D innovation plays a more vital role in promoting the growth of high-growth corporations. We further find that the corporate age primarily affects the relationship between R&D innovation and corporate growth. Young firms can gain higher upside returns through R&D innovation, but face more significant downside losses if innovation fails. We contribute to extant research by illuminating the moderating effect of corporate age on the process of R&D innovation on corporate growth. As the relationship between R&D innovation and political backgrounds has received increasing attention, further studies may be valuable for significantly advancing the current understanding of corporate growth at different growth rates and ages.

## Supporting information

S1 File Raw data. (XLS)

S2 File Measures the political background of corporate executives. (XLS)

S3 File Variable definitions. (XLS)

S1 Fig Trend chart by year. (TIF)
