# Supplementary figures and images for: Impact of R&D innovation and political background on corporate growth: A study based on private listed companies in China
Source: PLoS One. 2024 May 9;19(5):e0297329. doi: 10.1371/journal.pone.0297329 (PMC11081349; doi:10.1371/journal.pone.0297329)

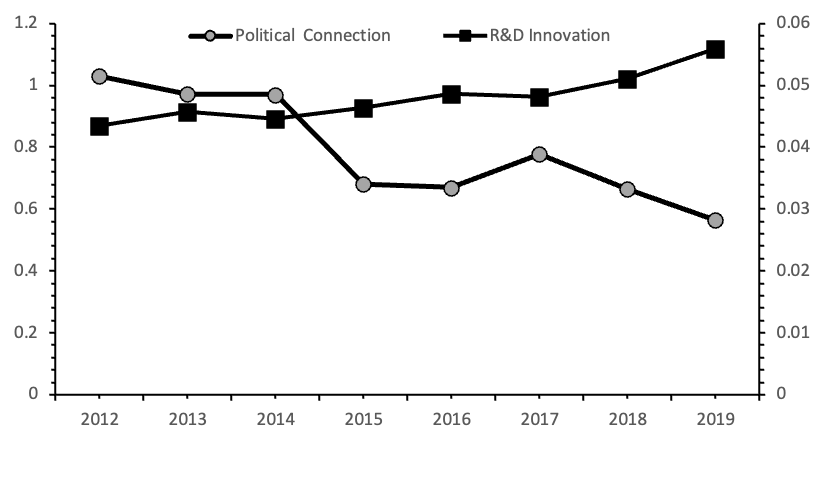

Supplement: S1 Fig — (TIF) [file pone.0297329.s004.tif]
